# Intussusception of the rectum in children; a rare case report 

**Published:** 2018

**Authors:** Pantea Tajik, Amir Hossein Goudarzian

**Affiliations:** 1 *Department of Pediatric Gastroenterohepatology, Amiralmomenin Hospital, Semnan, Iran.*; 2 *Student Research Committee, Mazandaran University of Medical Sciences, Sari, Iran. *

**Keywords:** Children, Rectum, Intussusception, Case report

## Abstract

A 28-month-old boy with hematochezia for 10 hours was admitted into our hospital. Colonoscopy was performed for the patient in which a mass-like lesion was seen with marron color. The mass suspected intussusception; thus, colonoscopy was interrupted and patient was sent to operation room. After 1 month the patient was good with no abdominal pain or defecation difficulty.

## Introduction

 Intussusception is a common cause of intestinal obstruction, afflicting one in 2000 infants and children (1-4), in which one segment of the gastrointestinal tract invaginates lumen of an adjacent intestinal segment. Yet most will be confined to the ileocolic distribution so the intussusception originates in the terminal ileum, advances through the ileocecal valve and often terminates near the hepatic flexure. Of note cases extending to the rectum or prolapsing through the anus have been reported (2-6).

Intussusception is most common among infants and toddlers and sixty percent of cases are seen in children younger than one year old, with a peak between 5-9 months of age. An infant with intussusception has sudden onset of crampy abdominal pain; the infant’s knees draw up, and the infant cries out and exhibits pallor with a colicky pattern occurring every 15 to 20 minutes and refuses to feed. As the intussusception progresses, and obstruction becomes prolonged, bilious vomiting becomes prominent, and the dilated, fatigued intestine generates less pressure and less pain (4-8).

The venous outflow from the intussusceptum is obstructed, leading to edema, weeping of fluid, and congestion with bleeding. Third space fluid losses and “currant jelly” stools will occur. Another unexpected feature of intussusception is lethargy. Between episodes of pain, the infant is glassy-eyed and groggy and appears to being sedated. A sausage-shaped mass caused by the swollen, intussuscepted bowel may be palpable in the right upper quadrant or epigastrium (6-8).

This study aims to introduce a 28 month old boy with hematochezia in whom colonoscopy showed that the small bowel intussuscepted up to rectum.

## Case Report

 A 28 month old boy having hematochezia for 10 hours, was admitted in our hospital. Mother of the infant explained that her son had two episodes of defecation with red blood-like string. She also mentioned that, although her son had allergy to yoghurt he ate yoghurt the day before. Vital signs were normal at admission. The physical examination and rectal exam was normal without blood. Abdominal examination did not reveal any abnormality or tenderness.

The lab test after admission; complete blood count (Hb 10 g/dl), urine analysis, liver function tests, blood urine nitrogen and creatinine were normal. In Stool exam 10 WBCs and 15 RBCs were reported. Accordingly, Ceftriaxone was ordered for the patient. After 8 hours the patient looked ill and the next Hb =7 g/dl was reported in the following complete blood count. Rectal bleeding was seen without defecation and rectal exam was seen. No abnormality was reported in abdominal sonography.

**Figure 1:**
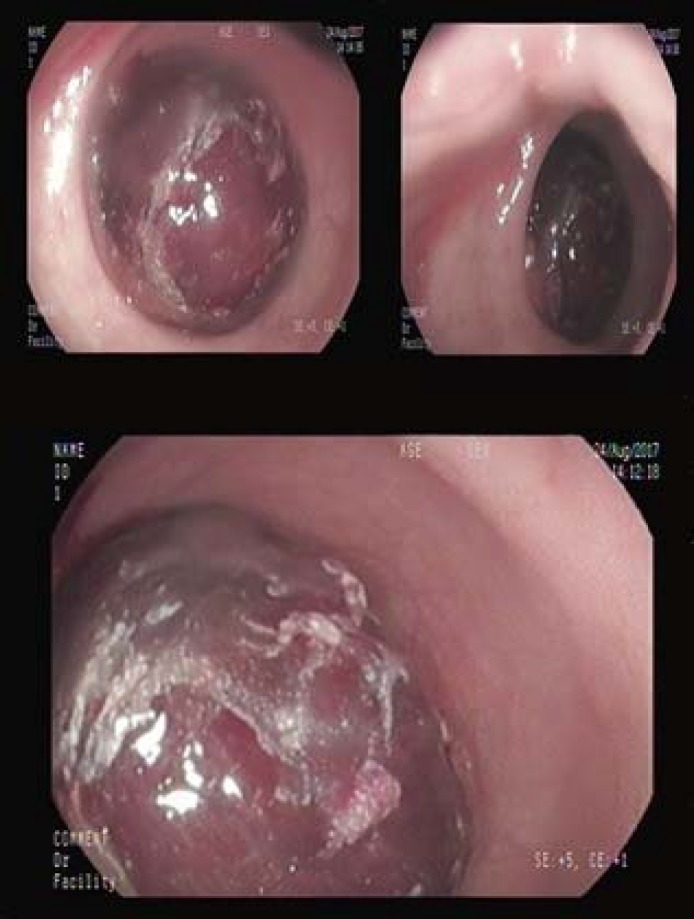
Ileum invaginated up to rectum seen in colonoscopy

 Colonoscopy was performed for the patient (figure 1). After insertion of colonoscope into anus a mass like lesion with marron color was seen in rectum. Intussusception was suspected; thus colonoscopy was interrupted and patient was sent to operation room. Patient was operated and ileocolic intussusception was seen up to rectum. The gangrened colon was resected up to ascending colon. After surgery patient was admitted to ICU and after 5 days he was well enough to go home. After 1 month the patient had no abdominal pain or defecation difficulty.

**Ethical issue**

 Informed consent was acquired from the parents of patient to publish this case report. 

## Discussion

Intussusception is the “telescoping” of a segment of proximal bowel (the intussusceptum) into downstream bowel (the intussuscipiens). Most cases occur in infants of 1 to 2 years old; in this age group, nearly all cases are idiopathic. Viral-induced lymphoid hyperplasia may produce a lead point in these children. In older children, the proportion of cases caused by a pathologic lead point increases. In young children, ileocolonic intussusception is common; the ileum invaginates into the colon, beginning at or near the ileocecal valve. When pathologic lead points are present, the intussusception may be ileoileal, jejunoileal, or jejunojejunal. As the intussuscepted bowel is pulled further and further into the downstream.

Intestine by motility, the mesentery is pulled with it and becomes stretched and compressed.The venous outflow from the intussusceptum is obstructed, leading to edema, weeping of fluid, and congestion with bleeding. Third space fluid losses and “currant jelly” stools result. Another unexpected feature of intussusception is lethargy. Between episodes of pain, the infant is glassy-eyed and groggy and appears to have been sedated. A sausage-shaped mass caused by the swollen, intussuscepted bowel may be palpable in the right upper quadrant (1-5). 

The diagnosis depends on the direct demonstration of bowel within-bowel which can be found simply by abdominal ultrasound. If the ultrasound is positive, or if good visualization could not be achieved, a pneumatic or contrast enema under fluoroscopy is indicated. This is a potentially useful way to both identify and treat intussusception. Air and barium can show the intussusception quickly and, when administered with controlled pressure, can reduce it completely most of the time. The success rate for pneumatic reduction is probably a bit higher. Hydrostatic reduction with barium approaches 90% if it is done when symptoms have been present for less than 24 hours. The pneumatic enema has the additional advantages over barium of not preventing subsequent radiologic studies and having no risk of causing barium peritonitis if perforation occurs. Non operative reduction should not be attempted if the patient is unstable or has evidence of pre-existing perforation or peritonitis (2-6).

Therapy must begin with placement of an IV catheter and a nasogastric tube. Before radiologic intervention is attempted, the child must have adequate fluid resuscitation to correct the often severe dehydration caused by vomiting and third space losses. Ultrasound may be performed before the fluid resuscitation is complete. Surgical consultation should be obtained early as the surgeon may prefer to be present during non-operative reduction. If pneumatic or hydrostatic reduction is successful, the child should be admitted to the hospital for overnight observation of possible recurrence (risk is 5% to 10%). If reduction is not complete, emergency surgery is required. The surgeon attempts gentle manual reduction but may need to resect the involved bowel after failed radiologic reduction because of severe edema, perforation, a pathologic lead point (polyp, Meckel diverticulum), or necrosis (7, 8).

So, in children with abdominal pain or rectal bleeding after taking history and performing physical examinations, abdominal sonography and radiography should be done. Intussusception is a cause of intestinal obstruction, rectal bleeding and abdominal pain. In our case intussusception was seen up to rectum and after surgery no complication was seen.

## Conflict of interests

The authors declare that they have no conflict of interest.
